# Supplementary material for: Βarriers and Gaps to Medical Care for Transgender Individuals: A TRANSCARE Scoping Review with a Focus on Greece
Source: Healthcare (Basel). 2024 Mar 13;12(6):647. doi: 10.3390/healthcare12060647 (PMC10970254; doi:10.3390/healthcare12060647)
Supplement: Supplementary file 1 [file healthcare-12-00647-s001.zip › Supplementary Table S1.pdf]

Supplementary Table S1. The detailed PubMed search strategy.

Search: ("delivery of health care"[MeSH Terms] OR ("delivery"[All Fields] AND "health"[All Fields] AND "care"[All Fields]) OR "delivery of health care"[All Fields] OR ("health"[All Fields] AND "care"[All Fields]) OR "health care"[All Fields]) AND ("barrier"[All Fields] OR "barrier s"[All Fields] OR "barriers"[All Fields]) OR ("access"[All Fields] OR "accessed"[All Fields] OR "accesses"[All Fields] OR "accessibilities"[All Fields] OR "accessibility"[All Fields] OR "accessible"[All Fields] OR "accessing"[All Fields]) AND ("transgender persons"[MeSH Terms] OR ("transgender"[All Fields] AND "persons"[All Fields]) OR "transgender persons"[All Fields] OR "transgender"[All Fields] OR "transgendered"[All Fields] OR "transgenders"[All Fields]) Filters: Free full text, in the last 5 years
