# Supplementary material for: Βarriers and Gaps to Medical Care for Transgender Individuals: A TRANSCARE Scoping Review with a Focus on Greece
Source: Healthcare (Basel). 2024 Mar 13;12(6):647. doi: 10.3390/healthcare12060647 (PMC10970254; doi:10.3390/healthcare12060647)
Supplement: Supplementary file 1 [file healthcare-12-00647-s001.zip › Supplementary Table S2.pdf]

Supplementary Table S2. General characteristics of documents identified from PubMed search (N=65) and other sources (N=4).

**Authors**	**Journal**	**Publication Year**	**Study type**	**Population/sample**	**Country/setting**	**Area/disease of focus**	**Barriers**	**Facilitators**
Goldenberg et al. (80)	Transgend Health	2020	Survey data	171 trans youth, 15-24 yo	USA	HC utilization	More positive sense of identity was associated with reduced difficulty accessing care for participants experiencing less stigma. Participants who experienced more anticipated stigma were less likely to use medical gender affirmation services.	Positive sense of identity, social support
Fung et al. (79)	Can Med Educ J	2020	Qualitative	11 medical residents	Canada	medical education	Lack of education, discomfort due to inexperience and lack of knowledge	
Ziegler et al. (78)	Transgend Health	2020	Qualitative	HCPs	Canada	PHC delivery	Lack of service coordination within organizations, lack of practitioner education	Continuing educational sessions, guidelines, and mentorship
Kcomt et al. (77)	SSM Popul Health	2020	Survey data	19,157 trans adults, 25-64 yo	USA	HC utilization	Discrimination, poverty, visual non-conformity	Having health insurance, disclosure of transgender identity
Dhillon et al. (76)	Am J Mens Health	2020	Scoping review	Trans men	Global	Cancer	Psychological discomfort and physical pain, sub-optimal patient-provider relationships, absence of a nonbinary approach to gender identity and health, lack of explanations, lack of sensitivity knowledge and experience, lack of cultural competence, including the use of appropriate pronouns, discrimination, “stereotypical” feminine/masculine aesthetics in HC services, challenges with health insurance	Establishing trusted relationship, accepting gender identity, safe and welcoming environment,

Lambrou et al. (75)	Transgend Health	2020	Qualitative	12 transmasculine adults, 18-35 yo	USA	HC delivery	Inadequate healthcare system that largely relies on essentialist, binary sex/gender framework, trans people being “forced” to accept a gender diagnosis, practitioners demonstrating “gatekeeping” power to withhold care	
de Santis et al. (74)	Perspect Psychiatr Care	2020	Mixed-methods	75 trans women	USA	HC delivery	Gaps in healthcare insurance coverage, personal financial issues, fragmented HC services (multiple doctors, different locations), lack of LGBTQI+ people in healthcare, refusal of services, discrimination, unstable relationships, poor communication with HCPs.	Treated as “regular” not "special clients, connection and communication with HCPs, mutually developed care plans, trust, provider willingness to be educated, employment of trans patient/client advocates and educators
Gamariel et al. (73)	PLoS One	2020	Qualitative	9 trans women and 18 cis MSM, 19-47 yo	Mozambique	HC access	Stigma and discrimination due to sexual identity, HIV-related stigma, gossip, breach of confidentiality in HC services	Peer educators with broad scope, social media as a means of engaging with health messaging
Kattari et al. (72)	SSM Popul Health	2019	Survey data	27,715 trans/nonbinary adults	USA	mental health	Individuals with mental health were significantly less likely to have a provider that treated them with respect and more likely to have needed to educate their provider on trans issues	
Zwickl et al. (70)	Int J Environ Res Public Health	2019	Online survey	928 trans adults	Australia	Needs assessment	Lack of trained HCPs, lack of gender services, lack of mainstreaming HC services in primary care	
Haviland et al. (71)	Oncol Nurs Forum	2020	Integrative literature review	LGBTQI+ adults	Global	Cancer care	Lack of cancer screening data and knowledge about screening guidelines by LGBTQI+Q populations and providers	Provider-created welcoming environments and caregiver inclusion

Yan et al. (84)	Infect Dis Poverty	2019	Qualitative	>14 trans women	China	HC utilization	Long and challenging identity search, stigma and discrimination, poor access to trans-specific services and unmet needs for mental health care, culturally-shaped expectations, low perceptions of HIV risk	
Griffin et al. (69)	Transgend Health	2019	Survey data	66 trans adults	USA	Needs assessment	Lack of mental health support and community stigma and personal financial barriers were associated with poorer health perceptions. Perceptions of lack of safety were associated with poorer health perceptions.	
Hines et al. (68)	J Assoc Nurses AIDS Care	2019	Qualitative	18 trans women, 21-60 yo	USA	HC utilization	Clinicians who don't understand, fragmented care, lack of insurance, low volume of transgender-competent clinicians	Provision of gender-affirming care, fostering patient engagement, performing appropriate health screenings, willingness to learn about transgender health.
Fauk et al. (67)	PLoS One	2019	Qualitative	29 trans women with HIV	Indonesia	HIV care	Limited availability of the services, limited simplicity and convenience of accessibility to services and discomfort felt while accessing the services	HCPs' positive attitudes during care provision, social relationships between trans patients and HCPs, proximity to healthcare facilities, free access to the services, information sessions on HIV infection and prevention
Luvuno et al. (66)	Afr J Prim Healthcare Fam Med	2019	Qualitative	9 trans adults	South Africa	Sexual/reproductive care	Hostile and discriminatory behavior by HCPs, no disclosure of trans identity, inability to provide care, violation of bodily privacy and confidentiality, treating trans patients as mentally unstable, doubts expressed by HCPs about trans identity, being made to conform	

							with assigned gender, unappealing service's environment, intimidating and offer religious behaviors	
Acosta et al. (65)	Psychiatr Q	2019	Qualitative	9 trans adolescents, 18 HCPs	USA	Psychiatric care	Incorrect use of preferred names/pronouns, legal name in electronic medical record as a barrier to engagement, HCPs uncertainty regarding the authenticity of patient's gender identity	Gaining understanding from patients as a resource to familiarize with terminology and process
Harb et al. (64)	Transgend Health	2019	Survey data	17 trans/genderqueer assigned female at birth adults	USA	Sexual/reproductive care	Lack of personal awareness about HPV, limited availability of competent care, distress about seeking sexual care, unappealing healthcare setting characteristics	HCPs' role and relationship
Jennings et al. (62)	Prev Med Rep	2019	National survey data	73 LGB, 25 trans, 1830 cis adults	USA	HC utilization	Trans adults were 2.76 times more likely to report poor quality of care and 2.78 times unfair treatment when receiving medical care than cisgender adults	
Breland et al. (63)	Transgend Health	2019	Mixed-methods	33 Trans/gender nonconforming youth, 29 caregivers	USA	HC utilization	Lack of accessible mental health providers, difficulty scheduling mental health assessment appointments, geographic distance, length of time between the readiness assessment and hormone initiation.	Respectful care delivery, consistent use of patients' preferred name and pronouns, presence of a care navigator
Cicero et al. (61)	ANS ADV Nurs Sci	2019	Integrative literature review	Trans adults	Global	HC utilization	Stigma, prejudice, discrimination, restricted health insurance benefits for medically necessary care, barriers to primary and preventative healthcare due to scarcity of available, knowledgeable, and affirming clinicians.	Social gender affirmation
Phillips et al. (60)	AIDS Care	2019	Survey data	890 young MSM and trans youth assigned male at birth	USA	HC utilization	Low awareness of available services was associated with how and where trans youth seek care, with 76% reporting this as their	

							primary reason for not seeking specific sexual health services.	
Velez et al. (59)	Transgend Health	2019	Survey data	52 trans/gender nonconforming adults	Puerto Rico	Social determinants of health	Lack of knowledgeable providers, discomfort during the encounter	
Frank et al. (58)	Transgend Health	2019	Survey data	273 young trans women	USA	Needs assessment	Avoiding healthcare due to cost and experiencing prior transgender-specific discrimination in a medical setting were associated with greater odds of having unmet healthcare needs.	
Stroumsa et al. (57)	Med Educ	2019	Online survey	223 primary care providers	USA	Medical education	Transphobia rather than education predicts provider knowledge of transgender health care.	
Harper et al. (56)	AIDS Patient Care STDS.	2019	Qualitative	66 trans/gender-diverse youth with HIV	USA	HIV care	Challenges in: confidentiality and privacy, service delivery, location of services, navigating the system, availability and awareness of services, discontinuity of care, negative provider interactions, instrumental support, disclosure of HIV status	
Marshall et al. (55)	Transgend Health	2018	Mixed-methods	96 trans and 28 cis adults	USA	Needs assessment	Challenges in: insurance coverage, access to and availability of transition-related care, and education of healthcare providers about trans issues.	
Shires et al. (54)	Ann Fam Med	2018	Survey data	308 primary care providers	USA	Care provision	Willingness to provide routine care decreased with provider age.	Willingness to provide Pap tests was higher among family physicians, those who had met a transgender person, and those with lower transphobia.
Kamen et al. (53)	Support Care Cancer	2019	Qualitative	273 LGBTQI+Q adults with cancer	USA	Cancer care	LGBTQI+ patients with cancer: are affected by providers' LGBTQI+-specific knowledge and skills, assumptions, and mistreatment,	

							negotiate disclosure of identities based on the safety of clinical encounters, have different experiences based on multiple intersecting identities, receive more effective care when members of their support networks are included	
Brumer et al. (52)	PLoS One	2018	Qualitative	14 trans adults	USA	Social determinants of care	Stigma and discrimination in healthcare settings, health-related information attained through social networks and online, gender identity, race and pervasive marginalization are key social determinants of transgender health	
Greene et al. (82)	PLoS One	2018	Survey data	1010 HC students	USA	Medical education	While 70-74% of respondents felt comfortable treating LGBTQI+ patients, fewer than 50% agreed that their formal training had prepared them to do so.	
Coutin et al.(81)	Can Med Educ J	2018	Survey data	556 medical residents	Canada	Medical education	Only 17% of participants predicted they would feel competent to provide speciality-specific trans-care by the end of their residency and only 12% felt that their training was adequate to care for this population.	
Fisher et al. (51)	LGBTQI+ Health	2018	Survey data	228 trans/nonbinary youth, 14-21 yo	Puerto Rico	HIV care	Stigma and confidentiality concerns: Nearly half of respondents had not disclosed their identity to their provider due to concern about an unaccepting provider. One-quarter were less inclined to discuss identity and sexual health with their provider due to concern that their provider would disclose this information to parents	Being out to parents about gender identity and having received gender-affirming hormone therapy.
Gahagan et al. (50)	Int J Equity Health	2018	Survey data	283 LGBTQI+ adults, 109 HCPs	Canada	PHC delivery	Uncertainty about the level of LGBTQI+-friendliness of their family doctor, their	Self-care, personal coping skills, self-esteem, safe and

							knowledge and cultural competence about, and the inclusiveness of the healthcare system. HCPs reported feeling discomfort when having to address LGBTQI+ specific issues with their patients, such as access to transition services for trans patients or family planning/reproductive health, mental health, domestic abuse and problematic drug use. Only 9.4% of HCPs indicated that they felt 'very knowledgeable' about issues related to gender identity/expression. HCPs identified the need for further education regarding LGBTQI+Q populations (e.g. CME LGBTQI+Q knowledge, communication skills, etc.), 43.4% considered inclusive signs and posters very important, and 49.1% considered language used in medical intake forms very important.	inclusive social environment, social support, access to LGBTQI+-friendly/safe spaces, community mental health resources
Beight et al. (49)	Transgend Health	2018	Qualitative	11 trans adults	Sweden	Mental health	Feeling objectification rather than subjectivity, need to confirm their identity rather than addressing mental health issues. Fearing the system (feelings of dependence and obligation, distrust, inflexibility of procedures, burden to behave in particular ways, lack of communication, focus on personal life rather than the problem, judgmental services, delays in care)	Including trans persons as advocates or as mentors into the care plans.
Heard et al. (48)	Peadiater Child Health	2018	Medical records/online survey	199 trans children/adolescents, 4.7-17.8 yo	Canada	Mental health	Adversity in healthcare settings, stress over long wait times for mental health services	Increasing HCPs' education on gender affirmative care, providing gender sensitivity training for HCPs, gathering preferred names and pronouns during triage, increasing visibility of support for LGBTQI++

								persons in clinics, increasing resource allocation to this field and creating policies so all healthcare settings are safe places
Lykens et al. (47)	LGBTQI+ Health	2018	Qualitative	10 genderqueer/nonbinary youth, 23-33 yo	USA	HC utilization	Providers approaching trans from a binary transgender perspective. Consequently, participants sometimes "borrowed" a binary transgender label to receive care, modified the healthcare they were prescribed, or went without healthcare, feeling disrespected and frustrated	
Puckett et al. (46)	Sex Res Social Policy	2018	Survey data	256 trans/gender nonconforming individuals, 16-73 yo	USA	HC utilization	Finances and insurance issues (pursue of hormone therapy, surgery, puberty blockers), a lack of service availability (and competent professionals), and fears or worries (anxiety being asked invasive questions, stigma, denial of care). systemic issues (problematic guidelines, unsafe environment) and incidents of bias within medical and mental health fields, as well as a lack of medical provider awareness and education. Other themes were interpersonal barriers (e.g., fears of rejection); age and need of parental consent for minors; other medical issues; and a lack of information about how to acquire care.	
Lee et al. (45)	Epidemiol Health	2018	National cross-sectional survey	278 trans adults	Korea	Transition-related care	Costs, negative experiences in healthcare settings, lack of specialized healthcare professionals and facilities, social stigma against transgender people.	

Gonzales et al. (44)	Milbank Q	2017	National survey	1443 trans/gender non-conforming and 314450 cis adults	USA	HC utilization	Transgender adults were more likely to be nonwhite, sexual minority, and socioeconomically disadvantaged compared to cisgender adults. Trans women were more likely to have no health insurance compared to cisgender women; transgender men were more likely to have no health insurance and no usual source of care; gender non-conforming adults were more likely to have unmet medical care needs due to cost and no routine checkup in the prior year.	
Clark et al. (43)	Fam Pract	2018	Cross-sectional survey	923 trans youth, 14-25 yo	Canada	PHC delivery	Levels of comfort with family doctor were negatively correlated with foregone mental healthcare in the previous 12 months, cost barriers, previous negative experiences with HCPs, uneducated HCPs about trans issues	Comfort with a family doctor was positively correlated with both general health and mental health status, as was having a doctor who was aware of one's transgender status.
Hughto et al. (42)	Transgend Health	2017	Online survey	364 trans adults	USA	Transition-related care	Younger age, low income, low educational attainment, private insurance coverage, and healthcare discrimination were significantly associated with being unable to access transition-related care	
Dowshen et al. (41)	Transgend Health	2017	Mixed-methods	25 trans women, 16-24 yo	USA	HC utilization	Lack of respect for or misunderstanding of gender identity, mismatch of mental health needs with available provider skills, challenges in finding HIV prevention services	Importance of workforce diversity, including representation of trans women in care teams.
Ross et al. (40)	Transgend Health	2016	Qualitative	10 transgender adults, HCPs, friends/family	Canada	HC utilization		Knowledge of trans issues, respect though the encounter, willingness to make referrals, connection/communication,

								listening, normalizing the transgender experience, ensuring support systems in place, helping in informed decision making, writing letters of support, letting transgender individuals take charge of their transition, self-educating, careful planning of one's healthcare journey, viewing healthcare as a do-it-yourself project
Porsch et al. (39)	Transgend Health	2016	Online survey	113 trans adults	USA	HC utilization		assurance that staff received trans sensitivity training (mean 3.8), the existence of gender identity nondiscrimination policies (mean 3.7), and the availability of transgender-specific services, such as hormone therapy (mean 3.7).
Rodriguez et al. (38)	Arch Sex Behav	2018	Survey data	6106 trans adults	USA	HC utilization	Being recognized as transgender to any extent had a significant effect on perceived discrimination in health care. Always recognized as transgender showed significant associations with social service and mental health settings, sex work and other street economy were also significantly associated with discrimination in health-care settings.	
Spencer et al. (37)	PLoS One	2017	Qualitative	12 HCPs	South Africa	Care provision	a small minority of healthcare providers offer gender affirming care, this is almost exclusively on their own initiative and is usually unsupported by wider structures and	

							institutions. The ad hoc, discretionary nature of services means that access to care is dependent on whether a transgender person is fortunate enough to access a sympathetic and knowledgeable healthcare provider.	
Hines et al. (34)	J Assoc Nurses AIDS Care	2017	Qualitative	18 trans women with HIV	USA	HIV care	Reluctance to face a diagnosis of HIV, concerns about lack of privacy and confidentiality, lack of support.	Psychosocial support, direct referrals from a provider, and guidance from a friend.
Muller (35)	BMC Int Health Hum Rights	2017	Qualitative	> 16 LGBTQI+ adults	South Africa	HC utilization	Lack of public health facilities and services, both for general and LGBTQI+-specific concerns, HCPs' refusal to provide care to LGBTQI+ patients, articulation of moral judgment and disapproval of LGBTQI+ patients' identity, forced subjection of patients to religious practices, lack of knowledge about LGBTQI+ identities and health needs, leading to poor-quality care. Delayed or avoided seeking healthcare in the past, without seeking out accountability or complaint mechanisms within the health system.	
Rossman et al. (34)	J Homosex	2017	Qualitative	206 LGBTQI+ youth, 18-27 yo	USA	HC utilization	Providers not asking about identity, internalized stigma, and belief that health and LGBTQI+Q identity are not related	
Johns et al. (33)	J Adolesc Health	2017	Survey data	250 trans women, 16-24 yo	USA	Social determinants of care	Having a history of unstable housing was associated with significantly higher odds of problems accessing both medical care and mental healthcare due to gender identity.	
Lavorgna et al. (30)	Mult Scler Relat Disord	2017	Online survey	307 LGBTQI+ adults	Italy	Multiple sclerosis	LGBTQI+ patients were associated with a smaller number of psychological consultations, compared to heterosexuals and more likely to change service compared to heterosexuals. The	

							number of service changes was associated with service friendliness and occurrence of homophobic behaviors.	
Reisner et al. (31)	AIDS Behav	2017	Mixed-methods	48 trans women, 19 HCPs	Peru	HIV care	Stigma, lack of provider training or guidelines on optimal trans care, service delivery obstacles (e.g., legal documents, spatial placement of clinics, hours of operation).	Hiring of TW staff
Logie et al. (30)	J Int AIDS Soc	2017	Qualitative	8 trans women, 18-30 yo	Jamaica	HIV care	HCP mistreatment, confidentiality breaches, and HIV-related stigma. Healthcare provider discrimination and judgment in HIV testing provision presented barriers to accessing HIV services (e.g. treatment), and resulted in participants hiding their sexual orientation and/or gender identity. confidentiality concerns (clinic physical arrangements that segregated HIV testing from other health services, fear that healthcare providers would publicly disclose their status, and concerns at LGBTQI+-friendly clinics that peers would discover they were getting tested). Anticipating HCP mistreatment if they tested HIV positive.	individual (belief in benefits of knowing one's HIV status), social (social support) and structural (accessible testing) factors that can increase HIV testing uptake.
McPhail et al. (29)	Can Med Educ J	2016	Qualitative	30 trans adults, 11 HCPs	Canada	Medical education	Lack of knowledge that resulted in a denial of trans-specific care and also impacted general care. Transphobia was identified as a barrier to quality care	
Stinchcombe et al. (28)	Geriatrics (Basel)	2017	Scoping review	LGBTQI+ older adults	global	General and end-of-life care	Health status, fear of discrimination and lack of trust, lack of knowledge and preparedness, cultural competence in the healthcare system.	social support and chosen family, intimacy,
Barrington et al. (27)	J Healthcare Poor Underserved	2016	Qualitative	26 MSM and trans women with HIV	Guatemala	HIV care	Stigma and discrimination due to non-normative gender expressions and / or sexual orientation. Retention-specific determinants	

							included HIV clinic dynamics and limited employment opportunities.	
Hughto et al. (26)	LGBTQI+ Health	2016	Survey data	5831 trans adults	USA	HC access	Being older, trans feminine, or a racial/ethnic minority, having low income and avoiding care due to discrimination were positively associated with care refusal	
Rocon et al. (25)	Cien Saude Colet	2016	Qualitative	15 trans adults	Brazil	HC access	Disrespect toward the adopted name, discrimination, and the diagnosis required for the gender reassignment process were major limitations to accessing the healthcare system.	
Safer et al. (6)	Curr Opin Endocrinol Diabetes Obes	2016	Brief literature review	Trans individuals	Global	HC access	Lack of sufficiently knowledgeable providers, financial barriers, discrimination, lack of cultural competence by providers, health systems barriers, and socioeconomic barriers.	
Albuquerque et al. (24)	BMC Int Health Hum Rights	2016	Systematic review	LGBTQI+ individuals	Global	HC access	Heteronormative attitudes imposed by HCPs, human rights violations in access to health services.	
Whitehead et al. (23)	PLoS One	2016	Survey data	LBGT adults	USA, rural	PHC utilization	Higher stigma scores were associated with lower utilization of health services for the transgender & non-binary group.	higher levels of disclosure of sexual orientation were associated with greater utilization of health services
Bauer et al. (22)	PLoS One	2015	Survey data	433 trans individuals, over 16 yo	Canada	PHC utilization	37.2% of transmasculine and 38.1% of transfeminine persons reported at least one trans-specific negative experience. Greater perceived physician knowledge about trans issues was associated with reduced likelihood of discomfort, and previous trans-specific negative experiences with a family physician with increased discomfort. Being previously married or having higher education associated	

							with increased risk of discomfort among transfeminine persons.	
Torres et al. (21)	BMC Pediatr	2015	Qualitative	11 HCPs of trans youth (ages 13-21)	USA	HC access	Lack of access to services, lack of social support, challenges in navigating HC system, limited HCPs' education	
Winter et al. (85)	Lancet	2016	Evidence review	Trans individuals	Global	Health, social, legislative issues	Transgender people live on the margins of society, facing stigma, discrimination, exclusion, violence, and poor health. They experience difficulties accessing appropriate health care, whether specific to their gender needs or more general in nature. Some governments are taking steps to address human rights issues and provide better legal protection for transgender people, but this action is by no means universal. The mental illness perspective that currently frames health-care provision for transgender people across much of the world is under scrutiny.	
ILGA-Europe (9)		2020	Report	LGBTQI+I	Europe	Human Rights		
Fantz et al. (2)	Clin Biochem	2014	Evidence review	Trans individuals	Global	HC access	Barriers: Stigmatization, structural and financial barriers, lack of experienced HCPs<br><br>Consequences: reluctance to disclose gender identity, consequences for long-term outcomes due to a lack of appropriate medical history including transition-related care. Even if a patient is willing to disclose their gender identity HC services lack the mechanisms necessary to collect and track this information.	
Giannou (83)	Durham theses,	2017	Qualitative	LGBTQI+ individuals	Greece	Health inequalities	a) Misinformation on health issues/needs	

	Durham University						b) Misdiagnosis<br><br>c) Exclusion e from preventive health care<br><br>d) Exclusion from sexual health information<br><br>e) Misreading of lesbianism as virginity<br><br>f) Exposure to inappropriate/ unsafe environments for disclosure<br><br>g) Exposure to offensive comments/jokes/ derogatory opinions on LGBTQI+ identities<br><br>h) Exposure to inappropriate questioning/ heterosexist assumptions<br><br>i) Invalidation of same-sex couples within health care<br><br>j) Lack of any acknowledgement of the challenges that LGBTQI+ carers face.	

Abbreviations: ***HC***: *health care,* ***PHC***: *primary healthcare,* ***HCP***: *healthcare professionals,* ***MSM***: *men who have sex with men,* ***LGBTQI+***: *lesbian, gay, bisexual, transgender*
